# Long-Term Trends in Urban Atmospheric Polycyclic Aromatic Hydrocarbons and Nitropolycyclic Aromatic Hydrocarbons: China, Russia, and Korea from 1999 to 2014

**DOI:** 10.3390/ijerph17020431

**Published:** 2020-01-08

**Authors:** Kazuichi Hayakawa, Ning Tang, Edward Nagato, Akira Toriba, Jin-Min Lin, Lixia Zhao, Zhijun Zhou, Wu Qing, Xiaoyang Yang, Vassily Mishukov, Andrey Neroda, Hae-Young Chung

**Affiliations:** 1 Low-Level Radioactivity Research Laboratory, Institute of Nature and Environmental Technology, Kanazawa University, O-24 Wake-machi, Nomi, Ishikawa 923-1224, Japan; n_tang@staff.kanazawa-u.ac.jp; 2 Graduate School of Life and Environmental Sciences, Shimane University, 1060 Nishitsugawa-machi, Matsue, Shimane 690-8504, Japan; nagato@life.shimane-u.ac.jp; 3 Institute of Medical, Pharmaceutical and Health Sciences, Kanazawa University, Kakuma-machi, Kanazawa, Ishikawa 920-1192, Japan; toriba@p.kanazawa-u.ac.jp; 4 Department of Chemistry, Tsinghua University, Beijing 100084, China; jmlin@mail.tsinghua.edu.cn; 5 Research Center for Eco-Environmental Science, Chinese Academy of Science, 18 Shuangging Road, Haidian District, Beijing 100085, China; zlx@rcees.ac.cn; 6 School of Public Health, Fudan University, No. 130 Dong Road, Shanghai 200032, China; zjzhou@shmu.edu.cn (Z.Z.); qingwu@fudan.edu.cn (W.Q.); 7 Chinese Research Academy of Environmental Science, No. 8 Anwai Beiyuan Dayangfang, Chaoyang District, Beijing 100012, China; yangxy@craes.org.cn; 8 V.I.II’ichiv Pacific Oceanological Institute, Far-Eastern Branch of Russian Academy of Science, 43 Baltiyskaya Street, 690041 Vladivostok, Russia; vmishukov@poi.dvo.ru (V.M.); aneroda@poi.dvo.ru (A.N.); 9 College of Pharmacy, Pusan National University, Gumijung-ku, Pusan 609-753, Korea; hyjung@pusan.ac.kr

**Keywords:** Far Eastern Asia, urban air, polycyclic aromatic hydrocarbons, nitropolycyclic aromatic hydrocarbons

## Abstract

Total suspended particulate matter (TSP) was collected during the summer and winter in five cities in China (Shenyang, Beijing, and Shanghai), Russia (Vladivostok), and Korea (Busan) from 1997 to 2014. Nine polycyclic aromatic hydrocarbons (PAHs) with four to six rings, including pyrene (Pyr) and benzo[*a*]pyrene (BaP), were determined using high-performance liquid chromatography with fluorescence detection. Two nitropolycyclic aromatic hydrocarbons (NPAHs), 1-nitropyrene (1-NP) and 6-nitrobenzo[*a*]pyrene (6-NBaP), were also determined using high-performance liquid chromatography with online reduction/chemiluminescence detection. Two Chinese cities, Beijing and Shenyang, showed very high concentrations of total PAHs (ΣPAH) and total NPAHs (ΣNPAH) with a large seasonal difference (winter > summer), although the concentrations decreased over time. In both cities, maximum mean concentrations of ΣPAH over 200 ng m^−3^ were observed in the winter. In Beijing, an increase in the ΣPAH concentration was observed in the winter of 2010, which was after the 2008 Beijing Olympic Games. The [1-NP]/[Pyr] ratio, a diagnostic parameter for source, was smaller in the winter than in the summer over the monitoring period, suggesting a large contribution of coal heating systems in the winter. In Vladivostok, concentrations of ΣPAH and ΣNPAH were lower than in the above two Chinese cities. The [1-NP]/[Pyr] ratio was larger than in the above Chinese cities even in the winter, suggesting that the contribution of coal combustion facilities, such as power plants for heating, was not very large. In Shanghai and Busan, concentrations of ΣPAH and ΣNPAH were much lower than in the above three cities. At the beginning of the monitoring periods, the [1-NP]/[Pyr] ratios, which were as large as those of Japanese commercial cities, suggested a large contribution from automobiles. After that, the contribution of automobiles decreased gradually. However, BaP concentrations were still over 1 ng m^−3^ in all cities monitored in China, Russia, and Korea, suggesting that the urban air pollution of PAHs and NPAHs in these regions should not be ignored.

## 1. Introduction

Northeastern Asia, including China, Far Eastern Russia, Korea, and Japan, is an area of continuing rapid industrial and economic development. This development is supported by large amounts of energy consumption. Excluding Russia, recent primary energy consumption in these countries occupied 28% of global consumption [1]. The fact that these countries have different energy sources is a major feature of this region: coal in China and Far Eastern Russia and oil in Korea and Japan. The combustion of coal, oil and biomass produces many kinds of pollutants, including carbon dioxide, sulfur oxides, nitrogen oxides, polycyclic aromatic hydrocarbons (PAHs), nitropolycyclic aromatic hydrocarbons (NPAHs), and particulates such as PM_10_ and PM_2.5_. With increases in energy consumption, large amounts of these pollutants are emitted into the atmosphere, and the composition of these pollutants differs by source.

Among these pollutants, the adverse effects caused by PM_10_ and PM_2.5_ that contain PAHs and NPAHs on the environment (globally) and human health are a large concern. The International Agency for Research on Cancer (IARC) has classified benzo[*a*]pyrene (BaP) and PM_2.5_ as Group 1 (carcinogenic to humans) and dibenz[*a,h*]anthracene, 6-nitrochrysene (6-NC), and 1-nitropyrene (1-NP) as Group 2A (probably carcinogenic to humans) [2,3]. Several NPAHs, such as 1,3-, 1,6-, and 1,8-dinitropyrene (DNP) and 1-NP, have shown very strong direct-acting mutagenicity [4,5]. Additionally, several oxidative metabolites of PAHs, such as hydroxy and quinoid derivatives of PAHs, have shown endocrine-disrupting and reactive oxygen species-producing activities [6,7].

We collected total suspended particulate matter (TSP) in several Japanese cities from 1997 to 2014 and determined PAH and NPAH concentrations [8]. Atmospheric concentrations of PAHs and NPAHs were high at the beginning of the monitoring period and then decreased gradually in commercial cities. The main contributors of these pollutants were automobiles in those cities, and emissions controls by the Japanese government for nitrogen oxides (NOx) and particulate matter (PM) from new cars were effective in decreasing the automobile contribution [9,10,11,12]. However, concentrations of PAHs did not decrease in a steel manufacturing city, where large amounts of coal were consumed in coke oven plants.

TSP samples were also collected in China (Shenyang, Tieling, and Fushung), Russia (Vladivostok), and Korea (Seoul), and PAH and NPAH concentrations were determined. The concentrations of PAHs in these cities were one or two orders of magnitude higher than those in Japanese cities, but the concentrations of NPAHs were not so high, suggesting a source difference [13,14,15,16,17,18]. For this report, we collected TSP samples in China (Shenyang, Beijing, and Shanghai), Russia (Vladivostok), and Korea (Busan) from 1999 to 2014 to clarify the recent trends in air PAH and NPAH pollution in terms of source.

## 2. Materials and Methods 

### 2.1. Sampling

**Figure 1:**
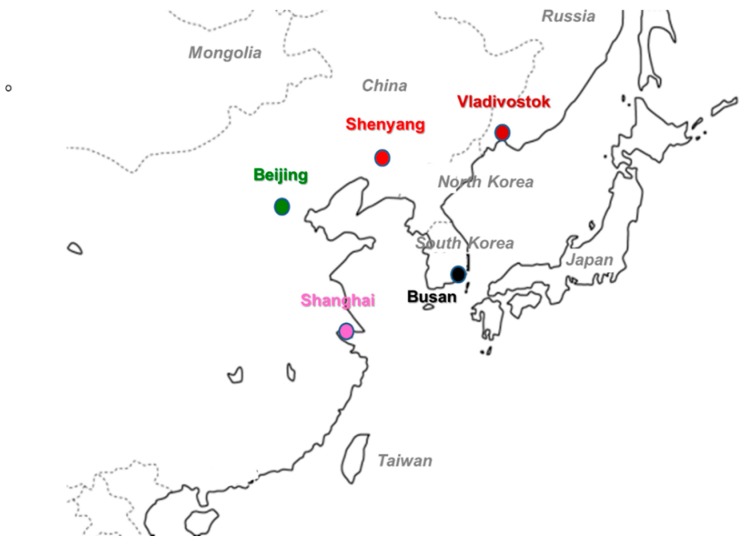
Airborne particulate sampling cities in East Asia.

TSP samples were collected in Shenyang, Beijing, Shanghai, Vladivostok, and Busan. Figure 1 shows a map of five cities in Far Eastern Asia. The populations of the five cities ranged between 600,000 and 24,000,000. Automobiles were the main means of transportation in all cities. Shenyang, Beijing, and Vladivostok used coal heating systems in the winter, while Shanghai and Busan did not. Other characteristics of the cities are listed in Table 1.

A high-volume air sampler equipped with a quartz fiber filter (8 × 10 inch, 2500QAT-UP, Tokyo Dylec, Tokyo, Japan) was set in a residential area not far from downtown as the representative site in each city, considering limitations in the number of samplers. TSP samples were collected for two weeks in the winter (January to February) and summer (July to August) at a flow rate of 1000 L/min. The filters were changed every day. After the filters were dried in a desiccator in the dark, they were stored at −20 °C until analysis. From the difference between the filter weights before and after sampling, TSP amounts were calculated. Other information on sampling has been described in previous papers [8,18].

### 2.2. Chemicals

The United States Environmental Protection Agency 610-PAHs mix, a mixture of 16 PAHs (including fluoranthene (FR), pyrene (Pyr), benz[*a*]anthracene (BaA), chrysene (Chr), benzo[*b*]fluoranthene (BbF), benzo[*k*]fluoranthene (BkF), BaP, benzo[*ghi*]perylene (BghiPe), and indeno[1,2,3-*cd*]pyrene (IDP)), were purchased from Supelco Park (Bellefonte, PA, USA). Two internal standards (pyrene-*d*_10_ (Pyr-*d*_10_) and benzo[*a*]pyrene-*d*_12_ (BaP-*d*_12_)) for PAHs were purchased from Wako Pure Chemicals (Osaka, Japan), and 1-NP, 6-nitrobenzo[*a*]pyrene (6-NBaP), and 2-fluoro-7-nitrofluorene (FNF, internal standard for NPAHs) were purchased from Chiron AS (Trondheim, Norway). The standard solutions of PAHs and NPAHs were prepared by dissolving each standard compound in ethanol. The internal standard solution was prepared by dissolving Pyr-*d*_10_ BaP-*d*_12_ and FNF in ethanol. All other chemicals used were of analytical reagent grade.

### 2.3. Sample Treatment and Analytical Procedures

The sample preparation method was the same as in previous reports [18,19] and is briefly described below. An area (2 × 5 cm) of each filter was cut into small pieces and put into a flask, and the internal standard solution was added to the flask. PAHs, NPAHs, and internal standards were twice extracted via sonication using a benzene/ethanol (3:1, *v*/*v*) solution. The solution was washed successively with sodium hydroxide and sulfuric acid solutions and then twice with ultrapure water. After filtering the organic solution with a membrane disk (HLC-DISK3, pore size 0.45 μm, Kanto Chemical Co., Tokyo, Japan), 100 μL of dimethyl sulfoxide (DMSO) was added to the filtrate. The mixture was concentrated to about 100 μL using a rotary evaporator, and the residual solution was dissolved in 900 μL of ethanol.

Nine PAHs were determined using a high-performance liquid chromatograph equipped with a fluorescence detector (HPLC-FLD, Shimadzu, Kyoto, Japan). The analytical column was a reversed-phase column (Inertsil ODS-P, 4.6 i.d. × 250 mm, GL Sciences Inc., Tokyo, Japan). The mobile phase was a mixture of acetonitrile/water with a gradient concentration mode. The flow rate of the mobile phase was 1 mL/min. The time program of the fluorescence detector was set to detect at the optimum excitation (Ex) and emission (Em) wavelengths for each PAH, as follows: Ex (nm), Em (nm) = 286, 433 (33.5–35.5 min; FR); 331, 392 (35.5–40 min; Pyr and Pyr-*d*_10_); 264, 407 (40–66.5 min; BaA, Chr, BbF, BkF, BaP, BaP-*d*_12_, and BghiPe); and 294, 482 (66.5–80 min; IDP). Two NPAHs, 1-NP and 6-NBaP, were determined by using a high-performance liquid chromatograph equipped with an online reducing column equipped with Pt/Rh and a chemiluminescence detector (HPLC-CLD, Shimadzu). The analytical column was a reversed-phase column (Cosmosil 5C18-MS-II, 4.6 i.d. × (250 + 150) mm, Nacalai Tesque, Kyoto, Japan). The mobile phase was a mixture of 10 mM imidazole buffer (pH 7.6)/acetonitrile (1:1, *v*/*v*), and the chemiluminescence reagent solution was an acetonitrile solution containing 0.02 mM *bis*(2,4,6-trichlorophenyl)oxalate and 15 mM hydrogen peroxide. The flow rate of the chemiluminescence reagent solution was 1 mL/min. Other conditions were the same as those in previous reports [8,18].

## 3. Results

### 3.1. Changes in PAH Concentrations over Time

**Figure 2:**
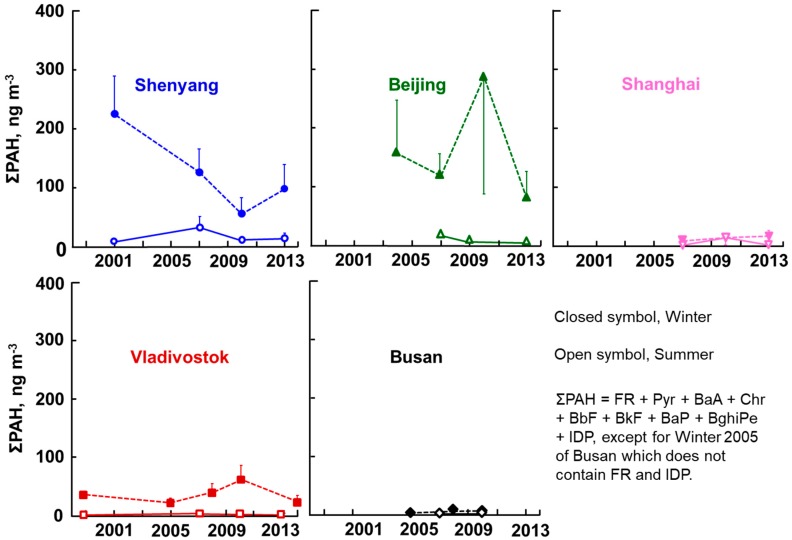
Atmospheric total polycyclic aromatic hydrocarbon (ΣPAH) concentrations.

The average atmospheric concentrations of nine PAHs and two NPAHs in five cities are listed in Tables S1–S5. Figure 2 shows the long-term changes in the summer and winter average ΣPAH concentrations of the five cities from 2001 to 2014, where each vertical bar means + or − the standard deviation (SD). Shenyang and Beijing showed very high concentrations with a large seasonal difference (winter > summer), followed by Vladivostok. The highest ΣPAH concentrations of Shenyang (223 ng/m^3^ in the winter of 2002) and Beijing (284 ng/m^3^ in the winter of 2010) were about 20 times higher than in Japanese commercial cities, such as Kanazawa, Sapporo, and Sagamihara, in the winter of 2013 [19]. A decreasing trend in ΣPAH concentrations was observed in Shenyang in the winter (statistical significance, *p* ≤ 0.01) and in Beijing in the summer, with a temporal increase in the winter of 2010. Vladivostok, whose ΣPAH concentrations were lower than those of the above two Chinese cities, did not show so clear a trend. Shanghai showed winter ΣPAH concentrations much lower than those of the above three cities, with smaller seasonal differences, suggesting that there was a big south–north difference in PAH pollution in China. Among the five cities, Busan showed the lowest winter ΣPAH concentration in 2010, but this was still more than three times higher than Japanese commercial cities [19].

### 3.2. Changes in NPAH Concentrations over Time

**Figure 3:**
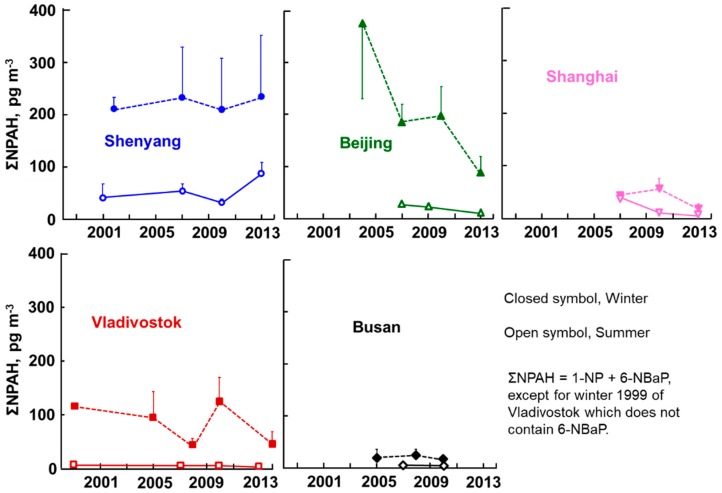
Atmospheric total nitropolycyclic aromatic hydrocarbon (ΣNPAH) concentrations.

The average atmospheric concentrations of 1-NP and 6-NBaP in the five cities are listed in Tables S1–S5. Figure 3 shows the long-term changes in the summer and winter average ΣNPAH concentrations of the five cities, where each vertical bar means + or − the SD. Shenyang, Beijing, and Vladivostok showed higher concentrations than did Shanghai and Busan, with a large seasonal difference (winter > summer). The winter ΣNPAH concentrations of Shenyang (208 pg/m^3^ in 2002) and Beijing (376 pg/m^3^) at the beginning of the monitoring period were as high as levels from the 1990s in Japanese commercial cities [19]. Beijing showed a decrease in ΣNPAH concentrations (*p* ≤ 0.01), and Vladivostok showed a decreasing tendency, but Shenyang did not. Shanghai and Busan showed much lower winter ΣNPAH concentrations than did the above three cities, with smaller seasonal differences. There was a south–north difference in the NPAH pollution that was similar to the PAHs.

### 3.3. PAH and NPAH Compositions

**Figure 4:**
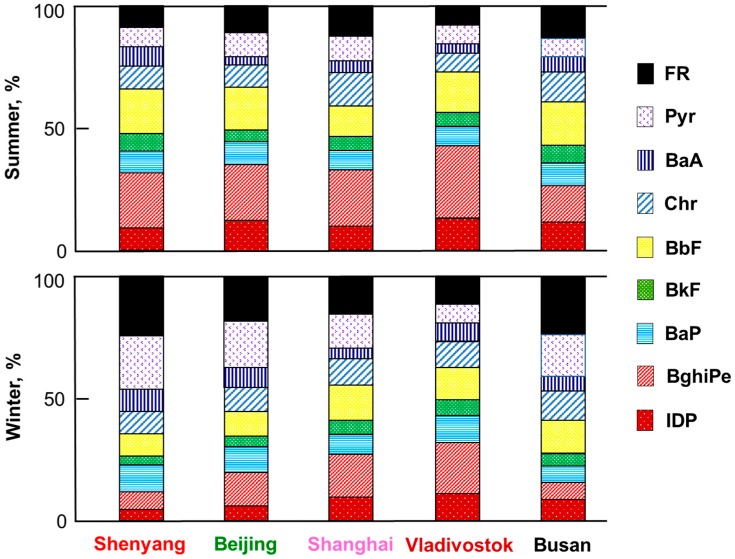
Composition of PAHs in 2013/2014.

Figure 4 compares the PAH compositions of TSP in the five cities in the summer and winter of 2013/2014. In every city, the fraction of four-ring PAHs (FR + Pyr + BaA + Chr), which accounted for the largest fraction among 4- to 6-ring PAHs, was larger in the winter than in the summer. It is known that the fraction of 4-ring PAHs in the gas phase increases with an increase in the atmospheric temperature [19]. This is the reason for the above seasonal differences. Among the five cities, the fraction of 6-ring PAHs (BghiPe + IDP) was the largest in Vladivostok in both seasons. 

**Figure 5:**
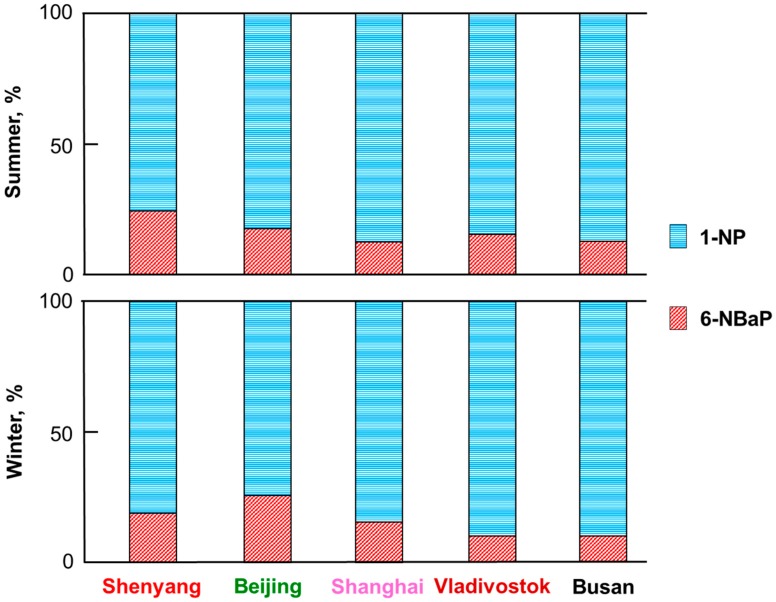
Composition of NPAHs in 2013/2014.

Figure 5 compares the NPAH composition of TSP in the five cities in the summer and winter of 2013/2014: 1-NP accounted for a much larger proportion (77.3–83.3% in summer, 74.8–84.5% in winter) than did 6-NBaP. The seasonal difference seen in Figure 4 was not observed in every city. The fraction of 6-NBaP was the largest in Beijing (25.2%) in the winter and in Shenyang (22.7%) in the summer. 

## 4. Discussion

### 4.1. Sensitive Determination of NPAHs

Amino derivatives of PAHs are highly sensitive to peroxyoxalate chemiluminescence reactions [20]. Therefore, we developed a high-performance liquid chromatograph equipped with an online reducing column packed with a Pt/Ph catalyst and a chemiluminescence detector [21,22]. This system has been used effectively for the determination of trace levels of NPAHs in airborne particulates [8,18]. This method was also used to determine 1-NP and 6-NBaP in this work.

### 4.2. Source Analysis

It is known that China is one of the most polluted (in terms of air) countries in the world. The poor atmospheric quality of Beijing became a significant concern for the Beijing Olympic Games held in 2008, and serious air pollution caused by extremely high PM_2.5_ concentrations became big global news in January 2013. The Chinese and Beijing governments took countermeasures such as the China Air Pollution Control Action Plan and the Coal Consumption Cap Plan, which included stopping the operation of factories, increasing traffic control, and reducing coal consumption (National Resources Defense Council, 2019). The overall decreasing trend in both ΣPAH and ΣNPAH concentrations (with a temporal increase in 2010 (Figure 2 and Figure 3)) suggests that the above countermeasures were effective in decreasing the atmospheric concentrations of both PAHs and NPAHs. 

The composition of PAHs in particulates is affected by emissions sources. The nitration of PAHs through combustion depends on temperature. The concentration ratios of NPAHs to corresponding PAHs ([NPAH]/[PAH]), such as [1-NP]/[Pyr] and [6-NBaP]/[BaP], were in the following decreasing order: wood burning (combustion temperature 500–600 °C) < coal burning (1100–1200 °C) < automobile engines (>2700 °C) [16,17]. Pyr and 1-NP were the best pair among the PAHs and NPAHs for a source comparison, since Pyr and 1-NP are major airborne particulate-bound PAHs and NPAHs (respectively). We have reported that [1-NP]/[Pyr] values were larger in Japanese commercial cities such as Tokyo, Sapporo, and Kanazawa than in Kitakyushu, a typical iron manufacturing city, where coke oven plans consume large amounts of coal [19]. Although the compositional patterns of PAHs and NPAHs in the Chinese, Russian, and Korean cities (Figure 4 and Figure 5) were not very different from the Japanese patterns, the concentration ratios of NPAHs to PAHs changed during the monitoring period.

**Figure 6:**
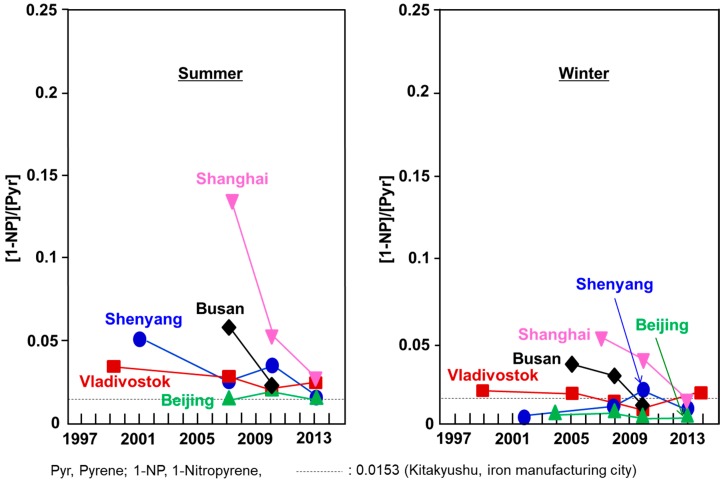
Diagnostic [1-NP]/[Pyr] ratios.

Figure 6 shows the long-term change of the [1-NP]/[Pyr] ratios in the cities. There was a seasonal difference (summer > winter) in all cities. Beijing and Shenyang showed smaller [1-NP]/[Pyr] ratios than did Shanghai and Busan, especially in the winter. The winter [1-NP]/[Pyr] ratios of the two cities were lower than was the ratio of Kitakyushu (0.0153) throughout the monitoring period. Kitakyushu is a typical iron manufacturing city in Japan, where a lot of coal is consumed in coke oven plants [19]. It has been reported that, in Beijing as well as in Shenyang, domestic coal heating systems emit large amounts of PAHs in the winter [18,21]. Considering these facts, the main contribution of pollution to Beijing and Shenyang can be attributed to coal heating systems but not to automobiles, although the registered numbers of automobiles are increasing. 

Vladivostok showed much higher ΣPAH and ΣNPAH concentrations than did the Japanese cities, but the concentrations were lower than in Shenyang and Beijing in both seasons (Figure 2 and Figure 3). In this city, there was a large increase in the construction of roads and bridges for the Asia Pacific Economic Cooperation (APEC) meeting held in 2012. This could have been a possible reason for the temporal increase in ΣPAH concentrations in 2010. The winter ΣNPAH concentrations slightly decreased in the winter during the monitoring period. The [1-NP]/[Pyr] ratios of Vladivostok were higher than those of Kitakyushu in the summer and lower in the winter (Figure 6), suggesting that the main contributors were automobiles in the summer and coal combustion in the winter.

Shanghai and Busan showed much lower ΣPAH and ΣNPAH concentrations, with larger [1-NP]/[Pyr] ratios, than did the above cities (Figure 6). Their largest [1-NP]/[Pyr] ratios (0.136 and 0.054, respectively) were in the summer of 2007. Afterwards, the ratios decreased dramatically. This decreasing trend in ΣNPAH concentrations and the [1-NP]/[Pyr] ratio was observed in Japanese commercial cities such as Tokyo, Sapporo, and Kanazawa during the period from 1997 to 2008 [8]. During this time, the Japanese government gradually and strictly reduced and controlled the levels of PM and NOx emissions from new automobiles and oil and automobile companies, improving oil and engine quality. These countermeasures decreased the emissions amounts of particulates and NOx from automobiles by a factor of around 1/10, and atmospheric PAH and PAH concentrations decreased in those cities. The decreasing rate of 1-NP concentration was larger than that of Pyr concentration in the atmosphere, because the main contributor to 1-NP is automobiles. Therefore, the [1-NP]/[Pyr] ratio decreased in the above Japanese commercial cities. The same reason is considered to be the reason for the decrease in the [1-NP]/[Pyr] ratio in Shanghai and Busan in Figure 6.

### 4.3. Comparison of Urban Air BaP Concentrations in Far Eastern Asian Countries

Environmental standards for atmospheric PAHs and NPAHs have not been globally set. However, several definitions for BaP environmental standards, such as reference values, setting values, and target values, have been recommended by several countries and organizations. The World Health Organization (WHO) has set target values at 1 ng m^−3^, which is based on the human lifetime cancer risk at approximately 1 × 10^−4^ [23]. WHO has also set the reference level for BaP concentrations at 0.12 ng m^−3^, calculating from the unit risk for lung cancer from PAH mixtures and the acceptable additional lifetime risk [24]. The European Environment Agency has set a target value for mean annual BaP concentrations (to minimize human health risk) at 1 ng m^−3^ [25]. China and New Zealand have also set the BaP environmental standard at 1 ng m^−3^ [26] and the BaP ambient air quality standard at 0.3 ng m^−3^ [27].

**Figure 7:**
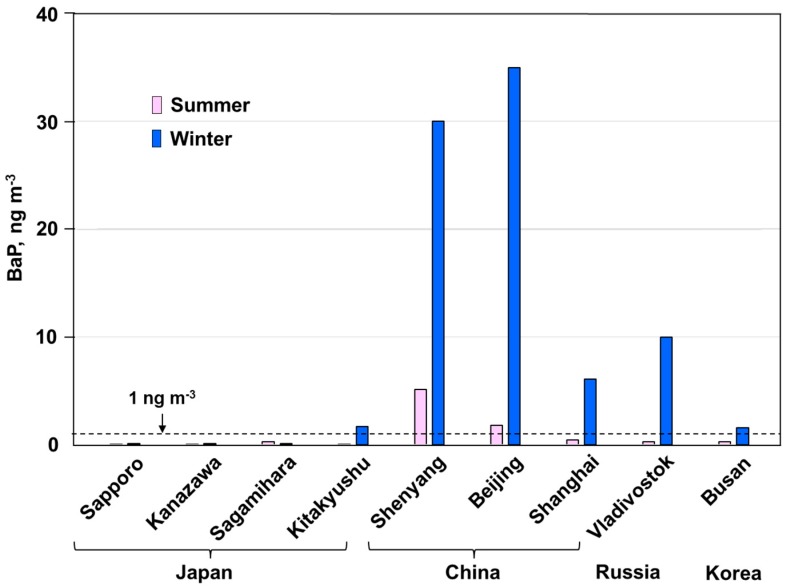
Benzo[*a*]pyrene (BaP) concentrations in Far Eastern Asian cities.

Figure 7 shows the urban atmospheric BaP concentrations of nine cities in Far Eastern Asian countries in the summer and winter of 2013–2014 except for Busan, whose sampling year was 2010. BaP concentrations of Japanese cities cited from our recent report [19] are used for the comparison. The BaP concentrations of Shenyang and Beijing were much higher than the Chinese and EU standards (1 ng m^−3^) in both seasons. The BaP concentrations of Shanghai, Vladivostok, and Busan were over the standards only in the winter. Even in Japan, Kitakyushu showed BaP concentrations over the standards in the winter. The BaP concentrations of all of the cities were over the WHO reference level (0.12 ng m^−3^) in both seasons. These results show that health risks cannot be neglected in any city in Far Eastern Asia. It is important to monitor the atmospheric PAHs and NPAHs in this region.

## 5. Conclusions

TSP was collected during the summer and winter in five cities in China (Shenyang, Beijing, and Shanghai), Russia (Vladivostok), and Korea (Busan) from 1997 to 2014, and nine PAHs and two NPAHs were determined. Two Chinese cities, Beijing and Shenyang, showed very high concentrations of ΣPAH and ΣNPAH, with a large seasonal difference (winter > summer) and maximum concentrations of ΣPAH of over 200 ng m^−3^ in the winter, although the concentrations decreased. The smaller [1-NP]/[Pyr] ratio suggests a large contribution from coal heating systems in the winter. In Vladivostok, concentrations of ΣPAH and ΣNPAH were high, but lower than those of the above two Chinese cities. The [1-NP]/[Pyr] ratio shows that the contribution of coal combustion facilities such as power plants to heating was not very large. In Shanghai and Busan, concentrations of ΣPAH and ΣNPAH were much lower than in the above three cities. The larger [1-NP]/[Pyr] ratios suggest that automobiles were the main contributor in those two cities. However, the contribution of automobiles decreased gradually. BaP concentrations were over 1 ng m^−3^ in many cities, suggesting that urban air pollution causing PAHs and NPAHs cannot be ignored in this region. 

## Figures and Tables

**Table 1:** Characteristics of sampled cities.

City	Location	Population	Temp. (Avg.), °C		Capital/Main Industry
	Latitude, Longitude		Summer	Winter	
Shenyang	41°37′ N, 123°25′ E	8,250,000	22.1	−12.1	Capital of Liaoning Province/the economic and industrial center of northeastern China
Beijing	39°54′ N, 116°24′ E	21,700,000	25.2	−2.4	Capital city of China/political center
Shanghai	31°13′ N, 121°27′ E	24,300,000	26.5	5.4	City under the direct control of the Chinese government/the economic, financial, and industrial center of China
Vladivosok	43°06′ N, 131°52′ E	600,000	16.8	−10.3	Capital of Primorsky Krai in Russia/a port city with shipping and fishing
Busan	35°06′ N, 129°02′ E	3,400,000	23.7	3.7	Korea’s second city/port city, which plays an important role in politics, the economy, and culture

## Supplementary Materials

The following are available online at https://www.mdpi.com/1660-4601/17/2/431/s1, Tables S1–S5: Variations of TSP, PAH and NPAH concentrations in Shenyang, Beijing, Shanghai, Vladivostok and Busan, respectively.
