# Supplementary material for: Long-Term Trends in Urban Atmospheric Polycyclic Aromatic Hydrocarbons and Nitropolycyclic Aromatic Hydrocarbons: China, Russia, and Korea from 1999 to 2014
Source: Int J Environ Res Public Health. 2020 Jan 8;17(2):431. doi: 10.3390/ijerph17020431 (PMC7013449; doi:10.3390/ijerph17020431)
Supplement: Supplementary file 1 [file ijerph-17-00431-s001.pdf]

Table S1. Variations of TSP, PAH and NPAH concentrations in Shenyang

Compounds	M.W.	2001/2002		2007/2008		2010		2013/2014	
		Summer	Winter	Summer	Winter	Summer	Winter	Summer	Winter
TSP (μg/m^3)		114 ± 29	194 ± 8.4	151 ± 37	171 ± 38	177 ± 17	171 ± 55	88 ± 36	178 ± 83
PAHs (pmol/m^3)									
FR	202.3	3.1 ± 1.2	260 ± 89	8.5 ± 2.5	67 ± 28	4.2 ± 1.9	33 ± 17	6.6 ±1.7	120 ± 57
Pyr	202.3	3.0 ± 0.81	250 ± 71	7.5 ± 1.7	74 ± 27	2.7 ± 0.92	37 ± 20	6.0 ± 1.6	110 ± 47
BaA	228.3	1.3 ± 0.41	110 ± 31	7.0 ± 2.9	68 ± 27	2.3 ± 0.69	32 ± 13	3.8 ± 1.1	35 ± 14
Chr	228.3	3.0 ± 1.1	140 ± 48	13 ± 6.2	83 ± 25	3.9 ± 0.85	40 ± 17	6.9 ± 1.5	45 ± 20
BbF	252.3	7.7 ± 3.7	79 ± 25	36 ± 27	74 ± 25	9.1 ± 1.7	24 ± 11	11 ± 3.6	37 ± 15
BkF	252.3	2.5 ± 1.2	38 ± 10	12 ± 8.2	29 ± 9.5	3.4 ± 0.72	12 ± 5.6	4.2 ± 1.1	15 ± 5.8
BaP	252.3	2.6 ± 0.84	78 ± 19	12 ± 5.7	49 ± 17	3.4 ± 0.81	24 ± 11	5.1 ± 0.99	30 ± 12
BghiPe	276.3	5.6 ± 3.9	48 ± 16	27 ± 13	57 ± 18	5.0 ± 0.75	19 ± 6.7	12 ± 3.3	37 ± 12
IDP	276.3	4.0 ± 2.3	34 ± 11	16 ± 8.3	28 ± 9.4	6.9 ± 1.4	19 ± 7.4	5.1 ± 0.75	18 ± 5.5
NPAH (fmol/m^3)									
1-NP	247.3	150 ± 100	720 ± 77	170 ± 41	790 ± 330	96 ± 28	730 ± 340	90 ± 25	760 ± 390
6-NBaP	297.3	11 ± 2.1	100 ± 27	36 ± 12	120 ± 56	21 ± 3.1	83 ± 41	22 ± 6.6	140 ± 72

Table S2. Variations of TSP, PAH and NPAH concentrations in Beijing

Compounds	M.W.	2004	2007/2008		2009/2010		2013	
		Winter	Summer	Winter	Summer	Winter	Summer	Winter
TSP (μg/m^3)		171 ± 58	757 ± 438	211 ± 58	96 ± 30	207 ± 125	125 ± 55	667 ± 152
PAHs (pmol/m^3)								
FR	202.3	160 ± 110	5.4 ± 1.6	110 ± 27	2.9 ± 0.54	310 ± 240	2.8 ± 1.4	77 ± 51
Pyr	202.3	170 ± 110	4.9 ± 1.3	100 ± 31	2.5 ± 0.49	290 ± 20	2.4 ± 1.2	81 ± 54
BaA	228.3	95 ± 57	2.8 ± 0.88	55 ± 20	0.84 ± 0.19	210 ± 170	0.91 ± 0.46	31 ± 20
Chr	228.3	92 ± 51	4.5 ± 1.6	45 ± 19	1.9 ± 0.53	190 ± 150	2.0 ± 0.94	38 ± 24
BbF	252.3	65 ± 40	10 ± 3.2	52 ± 20	4.9 ± 1.8	91 ± 53	3.5 ± 1.8	37 ± 23
BkF	252.3	28 ± 15	3.9 ± 1.1	18 ± 8.0	1.5 ± 0.39	38 ± 23	1.2 ± 0.60	14 ± 8.2
BaP	252.3	60 ± 35	5.3 ± 1.7	35 ± 16	2.0 ± 0.48	69 ± 41	1.8 ± 0.91	35 ± 17
BghiPe	276.3	48 ± 25	13 ± 4.0	60 ± 21	3.2 ± 0.80	26 ± 30	4.3 ± 2.2	43 ± 26
IDP	276.3	30 ± 19	7.2 ± 1.9	24 ± 10	3.2 ± 0.89	55 ± 36	2.3 ± 0.95	19 ± 12
NPAH (fmol/m^3)								
1-NP	247.3	740 ± 300	78 ± 20	680 ± 140	48 ± 20	440 ± 150	32 ± 11	260 ± 93
6-NBaP	297.3	650 ± 350	14 ± 3.5	41 ± 9.7	23 ± 14	320 ± 190	4.7 ± 2.1	73 ± 48

Table S3. Variations of TSP, PAH and NPAH concentrations in Shanghai

Compounds	M.W.	2007		2010		2013	
		Summer	Winter	Summer	Winter	Summer	Winter
TSP (μg/m^3)		84	107	81 ± 39	154 ± 42	34 ± 11	128 ± 44
PAHs (pmol/m^3)							
FR	202.3	1.2	3.3	0.76 ± 0.32	7.0 ± 3.8	0.94 ± 0.28	15 ± 8.3
Pyr	202.3	1.1	2.9	0.75 ± 0,31	5.6 ± 2.6	0.74 ± 0.21	13 ± 6.2
BaA	228.3	0.33	1.8	0.30 ± 0.093	5.0 ± 2.2	0.34 ± 0.17	4.1 ± 2.2
Chr	228.3	0.82	3.4	0.57 ± 0.22	9.2 ± 3.8	0.63 ± 0.17	10 ± 5.3
BbF	252.3	1.6	7.5	1.4 ± 0.37	9.8 ± 3.2	1.0 ± 0.38	11 ± 5.5
BkF	252.3	0.55	2.5	0.53 ± 0.14	4.1 ± 1.2	0.36 ± 0.14	4.4 ± 2.1
BaP	252.3	0.65	2.9	0.69 ± 0.19	5.7 ± 1.9	0.47 ± 0.18	6.1 ± 3.1
BghiPe	276.3	2.1	7.8	1.8 ± 0.49	9.0 ± 3.0	1.2 ± 0.50	12 ± 5.8
IDP	276.3	1.2	4.7	1.2 ± 0.37	5.9 ± 1.9	0.52 ± 0.23	6.6 ± 3.1
NPAH (fmol/m^3)							
1-NP	247.3	150	150	34 ± 12	200 ± 88	19 ± 5.1	170 ± 67
6-NBaP	297.3	5.1	20	7.4 ± 3.8	13 ± 3.6	2.3 ± 1.2	27 ± 10

Table S4. Variations of TSP, PAH and NPAH concentrations in Vladivostok

Compounds	M.W.	1999		2005	2007/2008		2010		2013/2014	
		Summer	Winter	Winter	Summer	Winter	Summer	Winter	Summer	Winter
TSP (μg/m^3)					95 ± 44	80 ± 22	41 ± 34	73 ± 33	50 ± 19	80 ± 18
PAHs (pmol/m^3)										
FR	202.3	0.49	30 ± 14	15 ± 4.1	1.1 ± 0.49	32 ± 8.9	1.1 ± 0.96	60 ± 18	0.33 ± 0.12	12 ± 6.4
Pyr	202.3	0.49	24 ± 13	16 ± 4.4	0.86 ± 0.34	26 ± 8.0	1.0 ± 0.81	52 ± 18	0.27 ± 0.098	8.1 ± 3.9
BaA	228.3	0.22	13 ± 10	6.5 ± 2.6	0.25 ± 0.091	10 ± 0.5	0.44 ± 0.30	26 ± 14	0.13 ± 0.071	6.7 ± 4.5
Chr	228.3	0.31	15 ± 9.9	10 ± 3.1	0.52 ± 0.17	13 ± 5.4	0.78 ± 0.50	32 ± 14	0.27 ± 0.11	9.6 ± 5.9
BbF	252.3	0.83	17 ± 10	11 ± 3.9	1.2 ± 0.60	18 ± 8.4	1.5 ± 0.92	31 ± 11	0.57 ± 0.26	12 ± 6.6
BkF	252.3	0.32	7.9 ± 5.1	5.7 ± 2.2	0.44 ± 0.23	8.7 ± 4.3	0.61 ± 0.39	15 ± 5.8	0.20 ± 0.10	6.0 ± 3.6
BaP	252.3	0.44	15 ± 10	9.6 ± 4.8	0.47 ± 0.41	16 ± 9.4	0.79 ± 0.57	25 ± 11	0.28 ± 0.099	10 ± 6.9
BghiPe	276.3	0.98	17 ± 12	8.6 ± 4.5	1.9 ± 1.4	24 ± 12	1.4 ± 0.99	12 ± 6.7	0.85 ± 0.31	16 ± 9.4
IDP	276.3	0.51	8.1 ± 7.0	10 ± 4.7	1.2 ± 1.2	18 ± 8.9	1.1 ± 0.86	20 ± 9.0	0.37 ± 0.15	8.1 ± 4.7
NPAH (fmol/m^3)										
1-NP	247.3	17	460 ± 230	290 ± 140	19 ± 2.5	350 ± 150	19 ± 9.7	340 ± 110	6.0 ± 3.9	150 ± 91
6-NBaP	297.3	8.7	N.D.	80 ± 84	3.1 ± 1.7	26 ± 18	4.4 ± 2.3	130 ± 74	1.4 ± 0.79	22 ± 15

Table S5. Variations of TSP, PAH and NPAH concentrations in Busan

Compounds	M.W.	2005	2007/2008		2010	
		Winter	Summer	Winter	Summer	Winter
TSP (μg/m^3)		44 ± 29	62 ± 16	39 ± 21	34 ± 7.3	50 ± 23
PAHs (pmol/m^3)						
FR	202.3	N.D.	0.40 ± 0.15	5.3 ± 2.7	0.57 ± 0.31	7.0 ± 3.2
Pyr	202.3	2.5 ± 1.4	0.37 ± 0.11	4.0 ± 2.6	0.54 ± 0.25	5.2 ± 2.4
BaA	228.3	1.1 ± 0.44	0.15 ± 0.056	1.9 ± 1.3	0.21 ± 0.12	1.5 ± 0.60
Chr	228.3	1.7 ± 0.62	0.27 ± 0.070	3.0 ± 1.6	0.41 ± 0.24	3.3 ± 1.1
BbF	252.3	1.9 ± 0.66	0.45 ± 0.12	2.7 ± 1.1	0.57 ± 0.27	3.2 ± 1.2
BkF	252.3	0.91 ± 0.36	0.17 ± 0.038	1.1 ± 0.47	0.21 ± 0.11	1.2 ± 0.41
BaP	252.3	1.9 ± 0.84	0.25 ± 0.056	1.8 ± 1.0	0.30 ± 0.17	1.6 ± 0.66
BghiPe	276.3	1.7 ± 0.75	0.55 ± 0.11	2.9 ± 1.4	0.43 ± 0.21	1.5 ± 0.56
IDP	276.3	N.D.	0.29 ± 0.033	1.7 ± 0.73	0.33 ± 0.16	1.9 ± 0.68
NPAH (fmol/m^3)						
1-NP	247.3	84 ± 47	20 ± 7.4	91 ± 44	12 ± 7.6	56 ± 22
6-NBaP	297.3	7.5 ± 3.8	0.87 ± 0.45	5.3 ± 1.6	2.0 ± 1.0	4.6 ± 2.8
